# Food Addiction in Patients with Newly Diagnosed Type 2 Diabetes in Northeast China

**DOI:** 10.3389/fendo.2017.00218

**Published:** 2017-08-30

**Authors:** Fan Yang, Aihua Liu, Yongze Li, Yaxin Lai, Guixia Wang, Chenglin Sun, Guang Sun, Zhongyan Shan, Weiping Teng

**Affiliations:** ^1^Department of Endocrinology and Metabolism, Endocrine Institute, Liaoning Provincial Key Laboratory of Endocrine Diseases, First Hospital of China Medical University, Shenyang, China; ^2^Department of Endocrinology and Metabolism, The First Hospital of Jilin University, Changchun, China; ^3^Discipline of Medicine, Faculty of Medicine, Memorial University, St. John’s, NL, Canada

**Keywords:** diabetes mellitus, type 2, food addiction, Yale Food Addiction Scale, obesity, thyroid-stimulating hormone

## Abstract

**Aim:**

The aim of the study was to explore the prevalence of food addiction (FA) in individuals with newly diagnosed type 2 diabetes mellitus (T2DM) in China and to analyze risk factors of FA.

**Methods:**

A total of 624 subjects [312 individuals with newly diagnosed T2DM, 312 age-matched and body mass index (BMI)-matched healthy participants] were recruited. All participants were asked to complete the Yale Food Addiction Scale (YFAS) and received physical and lab examinations. The T2DM group was further divided into a FA group and a non-FA group.

**Results:**

Of the patients with newly diagnosed T2DM, 8.6% (27/312) met the FA diagnostic criteria proposed by the YFAS (7.6% in men and 10.1% in women, *P* = 0.43), while 1.3% (4/312) met the criteria in the control group. Logistic regression analysis showed that FA in the T2DM group was positively related to BMI and negatively related to age. T2DM with FA had a significantly higher uric acid (UA).

**Conclusion:**

Both men and women with newly diagnosed T2DM, especially in northeast China, were more likely to suffer from FA. T2DM patients with FA were younger and had higher UA.

## Introduction

In 2013, the estimated prevalence rate of diabetes mellitus in China was 10.9%, while the prevalence rate of prediabetes was 35.7% (1). Type 2 diabetes mellitus (T2DM) accounts for more than 90% of overall diagnoses in China (2). According to the epidemiology, for 2007–2008 in China, the proportions of overweight and obese diabetic patients were approximately 40 and 25%, respectively (3). There is a large amount of metabolic similarity between obesity and diabetes. Overeating is one of clinical characteristics of T2DM. On the other hand, some individuals may have a compulsive relationship to certain foods and lose control over their ability to regular consumption (4). A growing number of evidence show similarities between overeating and substance use disorders, including biological mechanisms, potential commonalities in symptoms, comorbidities, behaviors, and personality characteristics (5). In 1956, Randolph defined the term food addiction (FA) in the scientific literature (6), which referred to individuals could not control food consumption rationally and had developed tolerance, withdrawal, cravings, and other behavioral characteristics that were similar to addictions to other substances. Most of convincing evidences of FA in animal model was that rodents showed addiction-like behaviors such as binging, withdrawal, and craving and neuronal changes similar to previous reports in human drug addicts such as downregulated striatal dopamine D2 receptors dopamine release in the mesolimbic dopamine system after a few weeks of intermittent access to sugar or high-fat food (7–9). FA seems to be involved in obesity and eating disorders. A meta-analysis indicated that compared to individuals with a healthy body mass index (BMI), the FA prevalence was doubled in the overweight/obese population (24.9 and 11.1%, respectively) (10). However, little research has been conducted regarding the relationship between T2DM and FA. We designed the study and aimed to find the prevalence of FA in individuals with new-found T2DM and biochemical characteristics between T2DM patients with and without FA who were recruited in Northeast China.

## Materials and Methods

### Participants

Three hundred and twelve patients (128 females, 184 males) with newly diagnosed T2DM were recruited from outpatients who visited the department of endocrinology and metabolism in the First Affiliated Hospital of China Medical University from February 2014 to January 2015. Additionally, 312 age- and BMI-matched control subjects (164 females, 148 males) with normal blood glucose were recruited from the department of endocrinology and metabolism in the First Affiliated Hospital of China Medical University and in the First Hospital of Jilin University. Individuals with past diabetes, impaired fasting glucose, impaired glucose tolerance, hyperthyroidism, and hypothyroidism at present or in the past, eating disorders associated with gastrointestinal disease, special eating patterns for pregnancy or operations within the past year, a psychiatric or eating disorder, or a major disease affecting their quality of life were excluded. T2DM patients were enrolled according to the 1999 WHO diagnostic criteria (11). All volunteers were asked to complete the Yale Food Addiction Scale (YFAS) questionnaire (Chinese version) as well as receive a physical and lab examinations. The subjects were classified as obese (BMI ≥ 28 kg/m^2^), overweight (24 ≤ BMI < 28 kg/m^2^), and normal/low weight (BMI < 24 kg/m^2^) based on the BMI criteria for Chinese individuals. Informed consent was received from all volunteers before they participated.

### Yale Food Addiction Scale

According to the substance dependence criteria in the Diagnostic and Statistical Manual IV TR, the YFAS was developed by Gearhardt and his colleagues to diagnosis FA (12). YFAS has been validated in a multitude of studies from different regions and different groups in recent years, including a Chinese population (12–15). A study of 950 students from normal school indicated the Chinese version of the YFAS had good validity (15). The scale was designed to assess FA through eight aspects of the participants’ eating behaviors in the last year. YFAS has two scoring options: one is FA diagnosis, and the other is the FA symptom score. Individuals are apportioned a FA symptom score in the range of 0–7. The FA diagnostic criteria is met when individuals endorse more than three symptoms and suffer from clinically significant impairment during the past 12 months (16). In this study, the subjects completed the questionnaire according to unified guidance. In this study, the prevalence, symptom scores of FA, the proportion of symptom counts ≥3, and the proportion of every symptom were compared between the T2DM patients and the healthy controls.

### Statistical Analysis

All statistical evaluations were completed using SPSS, version 16.0 (SPSS: Chicago, an IBM Company). Count data are presented as the mean values ± SDs. Maximum, minimum, and measurement data are expressed as percentages. For count data, the *t*-test and Rank sum test were conducted to examine differences. While comparing categorical variables, Fisher’s Exact Test and Chi-square test were used. Logistic regression analysis was applied to explore independent correlation factors for FA in individuals with newly diagnosed T2DM. *P* < 0.05 was considered to be statistically significant for all analyses in the study.

## Results

### Comparisons of the General Conditions between Patients Group and Control Group

The demographic and physical characteristics of volunteers are presented (Table 1). Between two groups, no differences existed in terms of age, BMI and TC (*t* = −0.86, 1.53, −0.72, all *P* > 0.05). T2DM participants had higher levels of height, weight, waist, weight-to-height ratio (WHtR), FBG, OGTT 2 h PG, TG, and LDL-C (*t* = 2.94 ~ 39.30, all *P* < 0.05). However, there were lower levels of uric acid (UA) and HDL-C (*t* = −3.51, −10.70, both *P* < 0.05) compared to normal controls.

**Table 1:** Differences in demographic and clinical characteristics between the group of newly diagnosed type 2 diabetes mellitus (T2DM) and the control group.

	T2DM Mean ± SD (min–max)	Control group Mean ± SD (min–max)	*t*	*P*
Number (men/women)	*N* = 312 (184/128)	*N* = 312 (148/164)		
Age (years)	48.27 ± 13.67	49.09 ± 10.03	−0.86	0.39
Height (cm)	167.64 ± 8.07	164.41 ± 7.84	5.01	<0.001
Weight (kg)	73.76 ± 13.80	69.56 ± 12.81	3.94	<0.001
BMI (kg/m^2^)	26.13 ± 3.79	25.65 ± 3.92	1.53	0.13
Waist (cm)	92.51 ± 10.42	85.25 ± 10.39	8.72	<0.001
WHtR	0.55 ± 0.06	0.52 ± 0.06	7.23	<0.001
FBG (mmol/L)	10.67 ± 3.65	5.20 ± 0.39	26.30	<0.001
OGTT 2 h PG (mmol/L)	19.02 ± 5.88	5.69 ± 1.14	39.30	<0.001
TC (mmol/L)	5.08 ± 1.21	5.32 ± 5.74	−0.72	0.47
TG (mmol/L)	2.54 ± 3.26	1.65 ± 1.94	4.14	<0.001
HDL-L (mmol/L)	1.09 ± 0.32	1.43 ± 0.47	−10.70	<0.001
LDL-C (mmol/L)	3.19 ± 0.93	2.99 ± 0.77	2.94	0.003
UA (μmol/L)	304.27 ± 101.89	332.40 ± 98.22	−3.51	<0.001
TSH (mIU/L)	2.01 ± 1.38	2.51 ± 2.02	−3.57	<0.001

*BMI, body mass index; WHtR, waist to height ratio; FPG, fasting plasma glucose; OGTT 2 h PG, OGTT 2 h plasma glucose; TC, total cholesterol; TG, triglyceride; HDL-C, high-density lipoprotein cholesterol; LDL-C, low-density lipoprotein cholesterol; UA, uric acid; TSH, thyroid-stimulating hormone*.

### Comparison of the Prevalence and Symptom Counts of FA between T2DM and Healthy Controls

The prevalence of FA in the patient group was 8.6% (27/312), while it was 1.3% (4/312) in normal controls, and there was significantly different between the groups (χ^2^ = 16.43, *P* < 0.001). The median symptom count scores were 1.3 ± 1.5 in patients group and 0.4 ± 0.9 in control group, and according to a rank sum test, the patient group had a significantly higher median for symptom counts (*Z* = −8.637, *P* < 0.05). Moreover, the proportion of participants who have a symptom count of more than 3 was 23.7% in T2DM patient group and 4.8% in healthy control group (RR = 4.93, *P* < 0.001). The most shared symptom accounted was “Persistent desire or repeated unsuccessful attempt to quit” (39.1%) (Table 2).

**Table 2:** Comparison of the results of Yale Food Addiction Scale (YFAS).

	Patient group	Control group	χ^2^	*P*
Number and prevalence of food addiction	27 (8.65%)	4 (1.28%)	16.43	<0.001
Median symptom count score	1.0	0.0	–	–
**Number and proportion of every symptom**				
Substance taken in larger amount and for a longer period than intended	97 (31.09%)	24 (7.69%)	54.64	<0.001
Persistent desire or repeated unsuccessful attempt to quit	122 (39.10%)	66 (21.15%)	23.87	<0.001
Much time/activity required to obtain, use, and recover	104 (33.33%)	23 (7.37%)	64.86	<0.001
Important social, occupational, or recreational activities given up or reduced	30 (9.62%)	4 (1.28%)	19.44	<0.001
Use continues despite knowledge of adverse consequences	18 (5.77%)	2 (0.64%)	11.62	<0.001
Tolerance	17 (5.45%)	0	–	<0.001
Withdrawal	18 (5.77%)	6 (1.92%)	6.24	0.012
Clinically significant impairment	28 (8.97%)	4 (1.28%)	17.43	<0.001

### Factors Related to FA in the Patient Group

According to the YFAS, the prevalence of FA was 8.6% (27/312) in individuals with newly diagnosed T2DM (in men and women, it was 7.6 and 10.1%, respectively) (Table 2). There was no relationship between the prevalence of FA and sex (RR = 1.33, *P* = 0.43). The average number of FA symptom scores in the food addicts from the patient group was 3.6 (SD = 0.8). Food addicts in T2DM group ranged in age from 20 to 57 years. When they were classified as 20–29, 30–39, 40–49, and 50–59 years based on age, the prevalence of FA was 34.78, 10.94, 7.58, and 7.87%, respectively. When food addicts were classified by BMI (under/normal weight, overweight, and obese), the prevalence of FA was 2.0, 5.7, and 20.4%, respectively. The prevalence of FA was positively correlated with BMI (RR = 2.87 between the overweight and the under/normal weight groups, RR = 10.33 between the obese and the under/normal weight groups, both *P* < 0.05). Among food-addicted subjects, 7.4% were under/normal weight, 25.9% were overweight, and 66.7% were obese. In addition, in the case group, there was no relationship between hypertension and the prevalence of FA (RR = 0.93, *P* = 0.86). As for food intake in T2DM patients with FA, men preferred high-fat and high-protein foods as well as sugary drinks, while women preferred sweets, salty snacks, and sugary drinks.

### Comparison between T2DM Patients with and without FA

All newly diagnosed T2DM subjects were split into the FA group (*n* = 27) and the NFA group (*n* = 285) based on the YFAS criteria (Table 3). The average age of the FA group was 11 years younger than the NFA group. In the T2DM group, all measurements related to obesity, diabetes, and metabolic syndrome (weight, BMI, waist, and WHtR), as well as UA, FPG, and HOMA-IR, were significantly higher in individuals with FA compared to those without FA (*t* = 4.96, 6.18, 4.53, 4.22, 2.57, 2.41, 3.08, all *P* < 0.05). In addition, in T2DM patients with FA, a trend of lower thyroid-stimulating hormone (TSH) levels was observed as well (*P* = 0.06). A logistic regression model was used to find the risk factors of FA in individuals with new-found T2DM. Waist, HOMA-IR, and UA were not the independent factors for FA. The prevalence of FA was associated with higher BMI (OR = 1.222, 95% CI: 1.051 ~ 1.421, *P* < 0.05) and younger age (OR = 0.953, 95% CI: 0.918 ~ 0.990, *P* < 0.05) as shown in Table 4.

**Table 3:** Comparison of biochemical measurements between newly diagnosed type 2 diabetes mellitus patients with and without food addiction (FA).

	FA Mean ± SD (min–max)	NFA Mean ± SD (min–max)	*t*	*P*
Number (men/women)	*N* = 27 (14/13)	*N* = 285 (170/115)		
Age (years)	38.04 ± 10.97	49.24 ± 13.52	−4.12	<0.001
Height (cm)	167.96 ± 7.93	167.61 ± 8.09	0.22	0.83
Weight (kg)	85.89 ± 17.63	72.61 ± 12.83	4.96	<0.001
BMI (kg/m^2^)	30.20 ± 4.40	25.74 ± 3.50	6.18	<0.001
Waist (cm)	100.93 ± 11.89	91.71 ± 9.93	4.53	<0.001
WHtR	0.60 ± 0.07	0.55 ± 0.06	4.22	<0.001
FBG (mmol/L)	12.28 ± 3.77	10.52 ± 3.61	2.41	0.027
OGTT 2 h PG (mmol/L)	19.91 ± 4.79	18.94 ± 5.98	0.82	0.414
HOMA-IR	8.37 ± 5.81	4.89 ± 2.67	3.08	<0.001
TC (mmol/L)	5.23 ± 1.09	5.06 ± 1.22	0.65	0.52
TG (mmol/L)	2.34 ± 1.45	2.55 ± 3.38	−0.32	0.75
HDL-C (mmol/L)	1.05 ± 0.22	1.09 ± 0.33	−0.81	0.42
LDL-C (mmol/L)	3.37 ± 0.97	3.17 ± 0.92	1.04	0.30
UA (μmol/L)	353.04 ± 119.64	299.74 ± 99.10	2.57	0.036
TSH (mIU/L)	1.65 ± 0.98	2.05 ± 1.41	−1.45	0.061

*BMI, body mass index; WHtR, waist to height ratio; FPG, fasting plasma glucose; OGTT 2 h PG, OGTT 2 h plasma glucose; HOMA-IR, homeostasis model assessment for insulin resistance index; TC, total cholesterol; TG, triglyceride; HDL-C, high-density lipoprotein cholesterol; LDL-C, low-density lipoprotein cholesterol; UA, uric acid; TSH, thyroid-stimulating hormone*.

**Table 4:** Logistic regression analysis of risk factors for food addiction in patients with newly diagnosed type 2 diabetes mellitus.

Variables	β	*P*	OR	95% CI
Age	−0.048	0.014	0.953	0.918 ~ 0.990
BMI	0.200	0.009	1.222	1.051 ~ 1.421
Waist	−0.002	0.942	0.998	0.942 ~ 1.057
HOMA-IR	0.115	0.055	1.122	0.997 ~ 1.271
UA	0.001	0.654	1.001	0.997 ~ 1.005

*BMI, body mass index; HOMA-IR, homeostasis model assessment for insulin resistance index; UA, uric acid*.

## Discussion

To the best of our knowledge, this was first study of its kind using the Yale FA Scale to investigate the prevalence of FA as well as the biochemical and metabolic characteristics of FA in a population with newly diagnosed T2DM. The correlation of FA and newly diagnosed T2DM was evaluated by the prevalence and symptom scores. The study found that the prevalence of FA was 8.6% in patients who were newly diagnosed type 2 diabetic patients, while only 1.3% of the healthy control population had FA in northeast China. The second finding is that T2DM patients with FA were younger and mostly in the age range of 20–29 years old. This result was different from reported data that overweight/obese women with over 35 years old may be more likely to suffer from food addiction (FA) (10). In logistic regression analysis, FA was positively related to BMI and was negatively correlated with age, but it had no relationship to sex or hypertension. The FA symptom count was higher in patient group. Moreover, the proportion of participants who had a symptom count of more than 3 was 23.7% in the patient group and 4.8% in the other group, which were significantly different (RR = 4.93, *P* < 0.001). The mean of the FA symptom count in the food addicts was 3.6 ± 0.8 in the patient group.

Food addiction symptom count was a method developed to evaluate FA. A meta-analysis showed that in eight studies, the weighted mean symptom score was 2.8 (SD = 0.4) (10), the minimum score was 1.8 (17), and the maximum score was 4.6 in obese subjects who were diagnosed with a binge eating disorder (BED) (16). Most studies suggested that individuals with a higher BMI should be more susceptible to FA (18, 19) and have higher symptom counts (17, 20). In addiction, clinical symptom counts were significantly correlated with obesity-related measurement indices including BMI, waist, hip circumferences, body weight, trunk fat, and body fat percentage (20).

There have been an increasing number of studies using YFAS to evaluate FA (21), the weighted average prevalence of FA in 20 research studies from a systematic review was 19.9% (10). However, the prevalence of FA varied widely among different regions, ethnic groups, and participants. In general, the FA prevalence in the entire population was lower compared to some particular samples. For example, the prevalence of FA was 5.4% in the Canadian province of Newfoundland and Labrador (20), and in the US, 5.8% of women in the research met the FA diagnosis criteria (18). Meanwhile, the prevalence of FA was exceedingly high in specific overweight and obese populations. A study in Italy including 112 adults seeking weight reduction treatment showed that 33.9% individuals met YFAS criteria for FA (22), whereas the FA prevalence rate was 15.2% in obese patients seeking weight loss interventions in another group (23). Meule reported that 41.7% of obese people looking for bariatric surgery (*n* = 96) received a FA diagnosis using the YFAS German version (14). Furthermore, quite a few studies chose subjects with BED as participants. Gearhardt found that the classification of FA was met by 41.5% of individuals with BED (*n* = 96) (24). BED patients seeking obesity treatment (*n* = 81) filled out the YFAS in other research studies, and 57% of them were food addicts (16). In our work, the prevalence of FA was 1.3% in individuals with normal blood glucose in northeast China, and 8.65% of new-found type 2 diabetes patients met the YFAS standards for FA. Currently, there are no studies on similar populations to compare the prevalence of FA. As far as we know, there were no researches studying the association between FA and insulin resistance. Our work found that HOMA-IR indicated differently between FA group and NFA group; however, according to the logistic text, it was not the independent factor for FA. Future studies in different regions are needed to assess the prevalence of FA and its characteristics in individuals with type 2 diabetes.

Whether sex will be one of the influencing factors of FA is still controversial. When a meta-analysis was conducted by sex in a systematic review, there was a higher mean prevalence rate in females of 12.2% compared to 6.4% in males (10). Eichen reported that there were no differences in sex between participants with and without FA in the research of 178 adults seeking weight loss treatment (23). In our work, the prevalence of FA in men and women was 7.6 and 10.1%, respectively. There was no significant difference between men and women. The reason was not clear, and a study in a large general Chinese population is needed to address whether there are any sex differences.

There was a significant negative correlation between TSH and alcohol craving (25). There also was a relationship between TSH and cocaine addiction (26). As for FA, the lower level of TSH, although not statistically significant in our work, should be discussed as well because it was consistent with the findings from the Newfoundland population (27). Several studies reported serum TSH is positively associated with BMI (28, 29). Moreover, the study in Newfoundland found that compared to obese non-food addicts, there was significantly lower serum TSH in obese food addicts (27). There might be a link between FA and the low level of TSH.

The study demonstrated that patients with new-found T2DM in northeast China had a higher prevalence of food addition, compared to volunteers with normal blood glucose. In the patient group, food addicts had younger ages, higher obesity measurements (weight, BMI, waist, WHtR), and higher levels of UA, FPG, and HOMA-IR. These results suggest that individuals who have a tendency for FA should regularly receive screening for diabetes and metabolic diseases.

Our current study also has some limitations that should be considered. This study did not record diet composition, smoking history, drinking history, and the medication history of food addicts in detail. In addiction, the age of participants is often within the 40–59 years old category, which might not accurately reflect possible correlations between FA and age. Additional research with samples that are more diverse and larger are needed to evaluate FA in China. Future research studies should explore the influence factors and the mechanism of FA to solve relative problems effectively.

## Ethics Statement

Informed consent was received from all volunteers before they participated. The research was conducted according to the guidelines provided in the Declaration of Helsinki, and the protocols were approved by the Medical Ethics Committee of the First Affiliated Hospital of China Medical University.

## Author Contributions

FY and AL wrote the main manuscript text, took part in designing the study, collected and analyzed the data. YL participated in data management and analysis. WT, YL, GW, and CS were involved in designing the research and data collection. GS wrote and reviewed the manuscript. ZS is the corresponding author, designed the study, interpreted the results, and contributed to manuscript revision.

## Conflict of Interest Statement

The authors declare that the research was conducted in the absence of any commercial or financial relationships that could be construed as a potential conflict of interest.
